# Human Non-Hypertrophic Nonunion Tissue Contains Osteoblast Lineage Cells and E-BMP-2 Activates Osteogenic and Chondrogenic Differentiation

**DOI:** 10.3390/cimb44110377

**Published:** 2022-11-09

**Authors:** Ryo Yoshikawa, Tomoaki Fukui, Keisuke Oe, Yohei Kumabe, Takahiro Oda, Kenichi Sawauchi, Kyohei Takase, Yuya Yamamoto, Yoshitada Sakai, Ryosuke Kuroda, Takahiro Niikura

**Affiliations:** 1 Department of Orthopaedic Surgery, Kobe University Graduate School of Medicine, Kobe 650-0017, Japan; 2 Division of Rehabilitation Medicine, Kobe University Graduate School of Medicine, Kobe 650-0017, Japan

**Keywords:** non-hypertrophic nonunion, bone morphogenetic protein-2, fracture healing

## Abstract

In this study, we examined the proliferation capability and osteogenic and chondrogenic differentiation potential of non-hypertrophic nonunion cells (NHNCs), and the effect of *Escherichia coli*-derived BMP-2 (E-BMP-2) on them. We enrolled five patients with non-hypertrophic nonunion. NHNCs isolated from nonunion tissue sampled during surgery were cultured, passaged, counted every 14 days, and analyzed. NHNCs were homogenous fibroblastic adherent cells and long-lived through at least 10 passages, with a slight decline. The cells were consistently positive for mesenchymal stem cell-related markers CD73 and CD105, and negative for the hematopoietic markers CD14 and CD45. NHNCs could differentiate into osteoblast lineage cells; however, they did not have strong calcification or sufficient chondrogenic differentiation capability. E-BMP-2 did not affect the proliferative capability of the cells but improved their osteogenic differentiation capability by increasing alkaline phosphatase activity and upregulating the gene expression of osterix, bone sialoprotein, and osteocalcin. E-BMP-2 enhanced their chondrogenic differentiation capability by upregulating the gene expression of aggrecan and collagen type II. We showed, for the first time, that NHNCs have the capacity to differentiate into osteoblast-lineage cells, although the chondrogenic differentiation potential was poor. Local application of E-BMP-2 with preservation of nonunion tissue is a potential treatment option for non-hypertrophic nonunion.

## 1. Introduction

The bone healing mechanism following fracture involves a complex interplay of mechanical and biological factors; 5–10% of fractures fail to heal, resulting in delayed union or nonunion [1]. Nonunion is difficult to treat and has a high financial impact [2]; the Weber and Cech classification [3] is widely used to classify various types. However, it is difficult to distinguish nonunion based on the classification. Recently, considering the ease of judgment in clinical settings, we defined nonunion without radiological callus formation including oligotrophic, comminuted (torsion-wedge, dystrophic, and necrotic), defect, and atrophic types in the Weber and Cech classification as non-hypertrophic nonunion [3,4]. Furthermore, we previously reported that the standardized uptake value on bone single-photon emission computed tomography is lower in non-hypertrophic than in hypertrophic nonunion [5].

Assessment of biological activity in a nonunion tissue is essential to develop effective treatment strategies for nonunion. We previously reported that tissue in hypertrophic nonunion contains multilineage mesenchymal progenitor cells [6], and osteogenic cells are expressed in pseudoarthrosis tissue [7]. However, no study has investigated the cells derived from non-hypertrophic nonunion tissues and methods for improving the differentiation capability.

Recombinant human bone morphogenetic protein-2 (rhBMP-2) is a well-known growth factor in bone regeneration owing to its high potency and ability to induce the osteogenic differentiation of osteoblasts and osteoblast precursors [8,9,10]. However, the production of large amounts of rhBMP-2 is expensive as most rhBMPs are mainly purified from mammalian sources, such as Chinese hamster ovary (CHO) cells [11]. To overcome this, *Escherichia coli*-derived BMP-2 (E-BMP-2) has been produced, at a cost that is expected to be low, using a molecular unfolding and refolding technique as an alternative to mammalian cells [12,13]. Yano et al. [14] have reported that E-BMP-2 has biological activity comparable to that of BMP-2 produced in CHO cells, while we previously demonstrated that E-BMP-2-loaded β-tricalcium phosphate granules effectively promote bone regeneration in long bone defects in vivo [15]. However, to the best of our knowledge, the effect of E-BMP-2 on nonunion cells has not been demonstrated.

Therefore, in this in vitro study, we focused on non-hypertrophic nonunion with the aim to examine the proliferation capability and osteogenic and chondrogenic differentiation potential of tissue-derived non-hypertrophic nonunion cells (NHNCs), and the effect of E-BMP-2 on them. The results of this study may help determine whether E-BMP-2 could be a potentially useful treatment option for non-hypertrophic nonunion.

## 2. Materials and Methods

### 2.1. Patient Characteristics

Nonunion of bone was defined as the failure of a fracture to heal within 6 months in a patient with no progressive bone repair radiographically within 3–6 months after the fracture [16,17]. Patients with infections, tumors, autoimmune diseases, or other systemic bone-related diseases and those who were administered hormones, steroids, vitamin D, or calcium were excluded from this study.

Non-hypertrophic nonunion was defined as nonunion without radiological callus formation including oligotrophic, comminuted (torsion-wedge, dystrophic, and necrotic), defect, and atrophic types in the Weber and Cech classification and was confirmed by three senior orthopedic trauma surgeons [3,5]. Five patients with non-hypertrophic nonunion surgically treated in our institution were enrolled (Table 1). The patient characteristics were as follows: mean age, 36.6 (range 20–59) years; sex, 3 male and 2 female patients; fracture sites, 1 tibial diaphysis, 1 clavicle diaphyseal, and 3 femoral diaphysis fractures. The initial treatments for original fractures consisted of conservative therapy for one patient, whereas the others underwent surgical treatments: intramedullary locking nail (three patients) and plate-and-screw fixation (one patient). The duration from the initial fracture to the surgery for nonunion was 6–12 (mean 9.9) months. The ethics committee of Kobe University Hospital approved this study (No. 1198), and informed consent was obtained from all patients before participation.

### 2.2. Isolation and Culture of NHNCs

NHNCs were isolated from nonunion tissues as previously described [6,7,18]. The central portion of a small number of nonunion tissues obtained during the surgeries was carefully dissected to avoid contaminating the bone, periosteum, and muscle, and then cultured. The nonunion tissues were resected, washed with phosphate-buffered saline (PBS; Wako, Osaka, Japan), minced into small pieces, and cultured in the original medium (Om), α-modified minimum essential medium (Sigma-Aldrich, St. Louis, MO, USA) containing 10% heat-inactivated fetal bovine serum (Sigma-Aldrich, St. Louis, MO, USA), 2 mM L-glutamine (Gibco BRL, Grand Island, NY, USA), and antibiotics, in a 100-mm culture dish.

The culture plates were incubated at 37 °C in a humidified atmosphere of 5% CO_2_. After 7 days of incubation, the culture dish was washed with PBS to remove nonviable cells and debris, and the culture medium was changed twice weekly. Approximately 2 weeks later, the adherent cells were harvested with 0.05% trypsin-0.02% ethylenediaminetetraacetic acid (Wako, Osaka, Japan) and passaged into culture flasks at a density of approximately 4 × 10^3^ cells/cm^2^ for further expansion. The cells from passages 3 to 4 were used in the following differentiation assays for each sample.

### 2.3. Preparation of E-BMP-2

E-BMP-2 used in this study was produced and provided by Osteopharma (Osaka, Japan), and was dissolved in PBS (Wako, Osaka, Japan) to 100 ng/mL according to the manufacturer’s recommendations. Details of E-BMP-2 production have been reported [14,19]. E-BMP-2 with a dimeric molecular structure was produced in human BMP-2 gene-transfected *E. coli* with a monomeric structure and stored in inclusion bodies that were collected [20]. The molecular structure was unfolded in protein-denaturing agents, and then refolded to form dimeric E-BMP-2 by removing the denaturing agents. Dimeric E-BMP-2 was subsequently purified using several chromatography steps.

### 2.4. Growth Kinetics

Passage 3 cells that had reached subconfluence were replated; after 2 weeks, the cells at passage 4 were passaged again. To examine long-term growth kinetics, the cells were counted using a hemocytometer every 14 days from passage 4 to passage 10.

Population doubling (PD), a method of calculating proliferative capability, was performed for each subculture using the following equation: PD = [log_10_(NH)-log_10_(N1)]/log_10_(2); where, N1 is the inoculum number, NH is the cell harvest number, and log is logarithm [21]. The calculated PD increase was added to the PD level of the previous passages to yield the cumulative PD level. First, NHNCs were maintained in the Om (Om group); then, to examine the effect of E-BMP-2 on proliferation, NHNCs were cultured in Om supplemented with 100 ng/mL E-BMP-2 (Om + BMP group). Histological images in both groups were visualized on day 7 at passages 4 and 10 using a BZ-X700 microscope (Keyence, Osaka, Japan).

### 2.5. Immunophenotyping of NHNCs Using Flow Cytometry

The surface antigen profiles of NHNCs at passage 3 or 4 were characterized using flow cytometry. In total, 4 × 10^5^ cells were incubated with the following phycoerythrin (PE)-conjugated anti-human antibodies: CD14, CD45, CD73, and CD105 (BD Biosciences, San Jose, CA, USA) for 60 min at 4 °C in the dark. Nonspecific mouse PE-conjugated immunoglobulin G (IgG; BD Biosciences) was used as an isotype control. After incubation, the cells were analyzed using the BD LSRFortessa flow cytometer (X-20; BD Biosciences). The data were analyzed using FlowJo software 10.7.1 (Treestar, Ashland, OR, USA) with a MAC^®^ workstation. The positive cell rate is expressed as percentage of fluorescence intensity greater than 1 × 10^3^.

### 2.6. Differentiation Studies

#### 2.6.1. Osteogenic Induction

To induce osteogenic differentiation, NHNCs were cultured for 28 days in an osteogenic medium consisting of the Om plus 10 nM dexamethasone (Sigma-Aldrich, St. Louis, MO, USA), 10 mM β-glycerophosphate (Sigma-Aldrich, St. Louis, MO, USA), and 50 mg/mL ascorbic acid (Wako, Osaka, Japan) [6]. Cells from passages 3–4 were used in the osteogenic differentiation assays. NHNCs were cultured in six-well plates at a density of approximately 4 × 10^3^ cells/cm^2^. NHNCs were maintained in osteogenic differentiation medium (Os group). Additionally, to examine the effect of E-BMP-2 on the osteogenic differentiation of NHNCs, the cells were cultured in osteogenic differentiation medium supplemented with 100 ng/mL E-BMP-2 (Os + BMP group).

Osteogenic differentiation was evaluated using Alizarin Red S (Hartman Leddon, Philadelphia, PA, USA) staining, alkaline phosphatase (ALP) activity assay, and real-time reverse transcription-polymerase chain reaction (RT-PCR) on days 7, 14, 21, and 28. Alizarin Red S staining was also used to assess mineralization in differentiated cultures. Histological sections stained with Alizarin Red S were also visualized using a BZ-X700 microscope. To quantitatively compare the mineralization of NHNCs, the cells stained with Alizarin Red S were destained with ethylpyridinium chloride (Wako, Osaka, Japan), the extracted stain was transferred to a 96-well plate (200 μL per well), and the absorbance at 562 nm was measured using a microplate reader, as previously described [6,22]. The cell layer from each well was sonicated using a Microson XL2000 ultrasonic cell disruptor (Misonix, Farmingdale, NY, USA) and stored at −80 °C until assayed for ALP activity by measuring the release of p-nitrophenol from p-nitrophenylphosphate, monitored using a SensoLyte pNPP ALP assay kit (AnaSpec Corp., San Jose, CA, USA). The protein concentration was standardized using a bicinchoninic acid protein assay kit (Pierce Biotechnology Inc., Rockford, IL, USA). The expression of the osteoblast-related genes runt-related transcription factor 2 (*RUNX2*), osterix (*OSX*), activating transcription factor 4 (*ATF4*), collagen type I (*COL1A1*), bone sialoprotein (*BSP*), and osteocalcin (*OCN*) was measured using real-time RT-PCR.

#### 2.6.2. Chondrogenic Induction

To induce chondrogenic differentiation, a three-dimensional pellet culture system was established for 21 days. Approximately 2.5 × 10^5^ cells in a 15-mL polypropylene tube were pelleted via centrifugation [6,23]. The cells were treated with chondrogenic medium consisting of high-glucose Dulbecco’s modified Eagles medium (Invitrogen, Carlsbad, CA, USA) with 100 nM dexamethasone, 50 mg/mL ascorbic acid, 0.4 mM proline (Sigma-Aldrich, St. Louis, MO, USA), 1% insulin-transferrin-selenium plus Premix (Sigma-Aldrich, St. Louis, MO, USA), and 10 ng/mL recombinant human transforming growth factor-β3 (R&D Systems, Minneapolis, MN, USA) [6,7,18,24]. NHNCs were maintained in chondrogenic medium (Ch group). Additionally, to examine the effect of E-BMP-2 on the chondrogenic differentiation of NHNCs, they were cultured in chondrogenic differentiation medium supplemented with 100 ng/mL E-BMP-2 (Ch + BMP group).

Chondrogenic differentiation was evaluated using Safranin-O (Chroma, Munster, Germany) staining and real-time RT-PCR on day 21. Safranin-O staining was also used to assess the general morphology and proteoglycan content in cartilaginous tissues. For histological assessment, the pellets were embedded in paraffin, sectioned, and visualized using a BZ-X700 microscope. The expression of chondrocyte-related genes aggrecan (*ACAN*), collagen type II (*COL2A1*), collagen type X (*COL10A1*), and Sry-type high-mobility group box 9 (*SOX9*) was measured using real-time RT-PCR.

#### 2.6.3. Isolation of Control Cells Using a Reamer-Irrigator-Aspirator (RIA)

For Alizarin Red S and Safranin-O staining, positive control cells were isolated using the RIA system, a relatively new tool for harvesting autologous bone grafts by reaming the intramedullary canal of long bones [25]. This system has been reported to contain mesenchymal stromal cells (MSCs) with high osteogenic and chondrogenic potency [26,27]. The cells were cultured under the same conditions as the NHNCs without E-BMP-2.

#### 2.6.4. Real-Time RT-PCR Analysis

Total RNA was isolated from each sample using the RNeasy mini kit (Qiagen, Valencia, CA, USA) according to the manufacturer’s instructions. The total RNA was reverse transcribed to single-strand complementary DNA (cDNA) using a high-capacity cDNA RT kit (Applied Biosystems, Foster City, CA, USA). The converted cDNA samples were amplified using PCR with Taq Gold DNA polymerase (Applied Biosystems). Measurements were performed in duplicate using an Applied Biosystems 7500 real-time PCR system and the primers were purchased from Thermo Fisher Scientific Inc. (Waltham, MA, USA) (Table 2). Real-time RT-PCR was performed using a thermal cycler (Programme Temperature Control System PC-707; ASTEC, Fukuoka, Japan). The housekeeping gene glyceraldehyde 3-phosphate dehydrogenase (*GAPDH*) was analyzed to monitor RNA loading. NHNCs in the Os group on day 7 and the Ch group were used as controls.

### 2.7. Statistical Analysis

Data were analyzed using the statistical package for the social sciences (SPSS) 22.0 (IBM Japan, Tokyo, Japan). Kruskal–Wallis test and Dunn–Bonferroni post hoc test were used to compare weekly differences in the absorbance of Alizarin Red S staining and ALP activities in the Os and Os + BMP groups. To determine whether there were significant differences in PD, absorbance of Alizarin Red S staining, ALP activity, and real-time RT-PCR results between the groups (Om vs. Om + BMP, Os vs. Os + BMP, and Ch vs. Ch + BMP), we performed the Mann–Whitney *U* test. Differences were considered statistically significant at *p* < 0.05.

## 3. Results

### 3.1. Growth Kinetics and Morphological Characteristics

**Figure 1:**
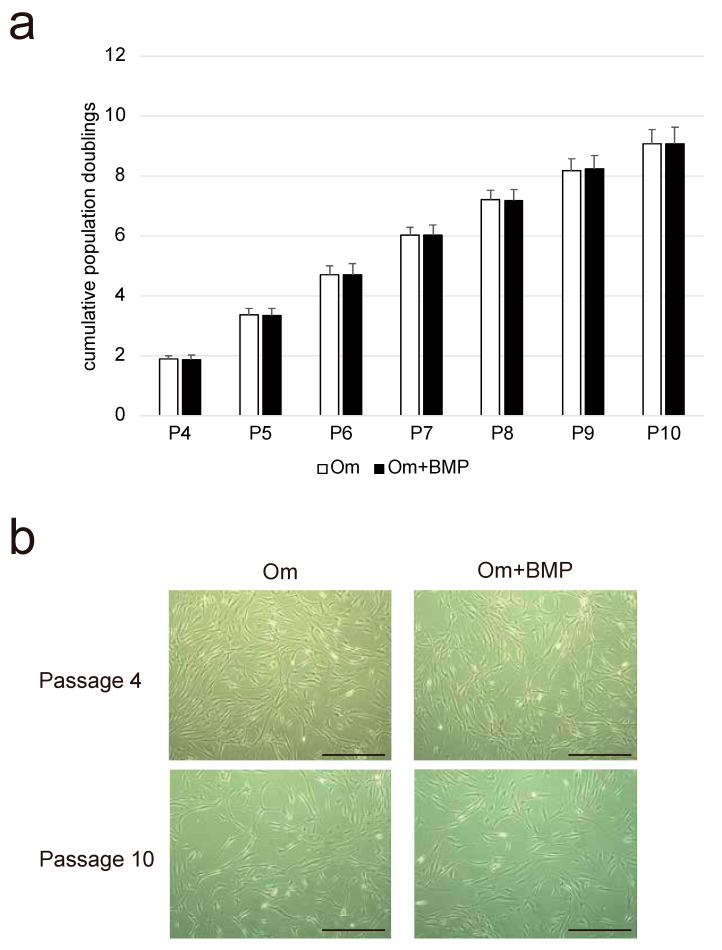
Evaluation of the proliferative capability of non-hypertrophic nonunion cells (NHNCs). (**a**) Cumulative population doubling (PD) values were determined for each subculture of NHNCs in original medium (Om) and Om with E-BMP-2 (Om + BMP). (**b**) Phase-contrast images of adherent NHNCs with Om and Om + BMP displaying fibroblastoid morphology at passages 4 and 10. Scale bar = 500 μm.

The cells showed longevity in culture and sufficient capacity to expand. The calculated PD suggested that the NHNCs could be cultured through at least 10 passages with a slight decline in proliferation rates (Figure 1a). In addition, there was no difference in proliferation capability between the Om and Om + BMP groups at each subculture stage. All NHNCs at passages 4 and 10 in both groups had similar fibroblast-like spindle-shaped morphology and the cell density was reduced with each passage (Figure 1b). E-BMP-2 had no influence on the appearance of NHNCs.

### 3.2. Immunophenotypes

**Figure 2:**
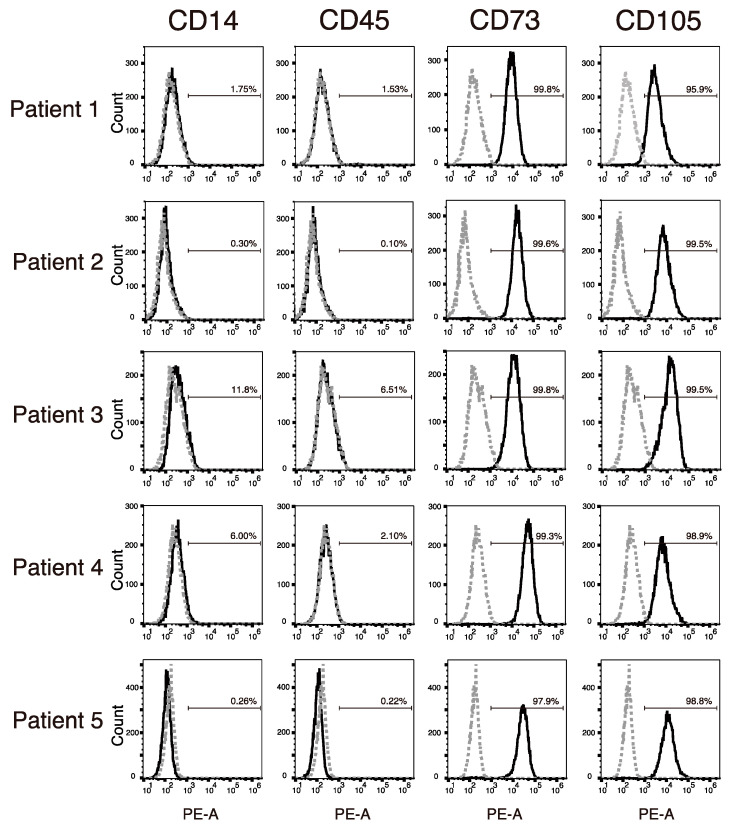
Flow cytometric analysis of the expression of cell surface markers of non-hypertrophic nonunion cells (NHNCs). Gray dotted lines are isotype control. Positive percentage of the cell surface is shown in each histogram.

Flow cytometric analysis showed that the NHNCs derived from all patients were negative for the hematopoietic stem cell markers CD14 and CD45, and positive for the mesenchymal stem cell markers CD73 and CD105 (Figure 2). The positive expression rate (mean ± standard deviation) of CD14, CD45, CD73, and CD105 was 4.02% ± 4.94%, 2.09% ± 2.61%, 99.3 ± 0.80%, and 98.5% ± 1.50%, respectively.

### 3.3. Osteogenic Differentiation Potential

**Figure 3:**
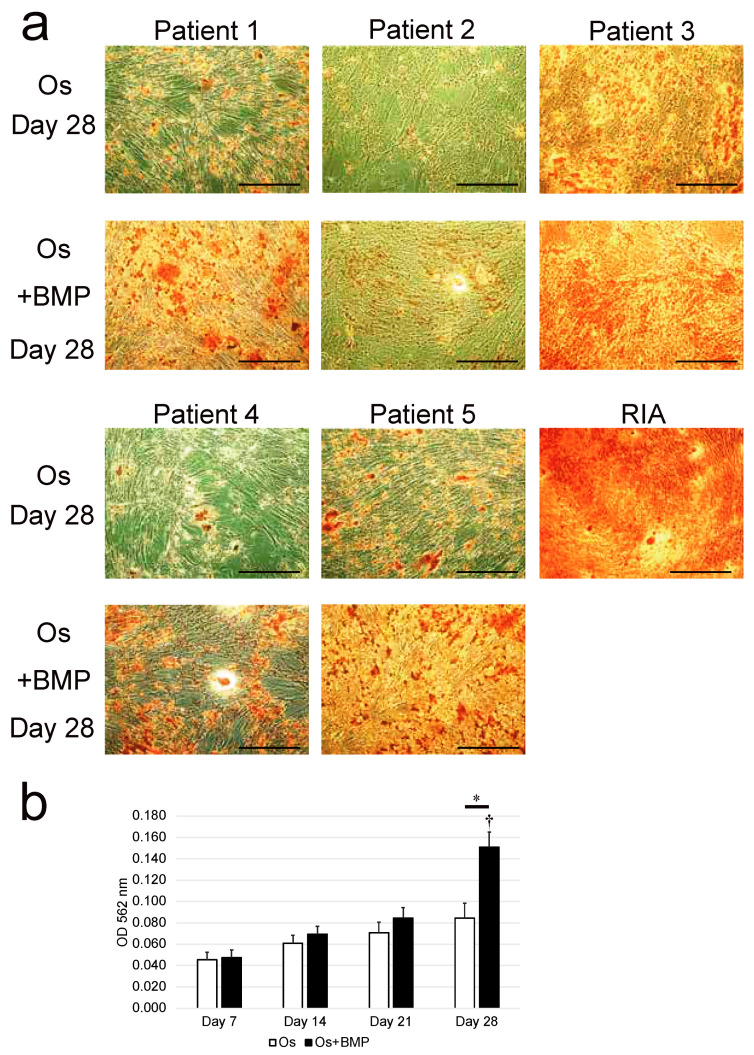
Analysis of the calcification capability of non-hypertrophic nonunion cells (NHNCs). (**a**) Histological analysis of the osteogenic capability of NHNCs and cells derived from a reamer-irrigator-aspirator (RIA, positive control) stained using Alizarin Red S after 28 days of incubation in osteogenic medium (Os) and Os with E-BMP-2 (Os + BMP). In Os, a slightly mineralized matrix was observed as a red-stained nodule. In Os + BMP, mineralization nodule formation was more prominent. Scale bar = 500 μm. (**b**) Mineralization activity measured as absorbance of NHNCs in Os and Os + BMP at 562 nm on days 7, 14, 21, and 28. * *p* < 0.05 in the indicated groups and ^†^ *p* < 0.05 compared with day 7 results in Os + BMP group.

After 28 days of incubation under osteogenic conditions (Os group), the NHNCs formed a slightly mineralized matrix, observed as a nodule stained red by Alizarin Red S (Figure 3a). In addition, mineralized bone nodule formation was more prominent in the Os + BMP group than in the Os group. Quantification of the Alizarin Red S staining intensity revealed that mineralization in the NHNCs increased with time in both groups. However, there were no significant weekly differences in the Os group (*p* = 0.068). In contrast, in the Os + BMP group, the mineralization on day 28 was significantly higher than that on day 7 (*p* = 0.001). In addition, the mineralization activity of the Os + BMP group on day 28 was significantly higher than that of the Os group on day 28 (*p* = 0.016) (Figure 3b).

**Figure 4:**
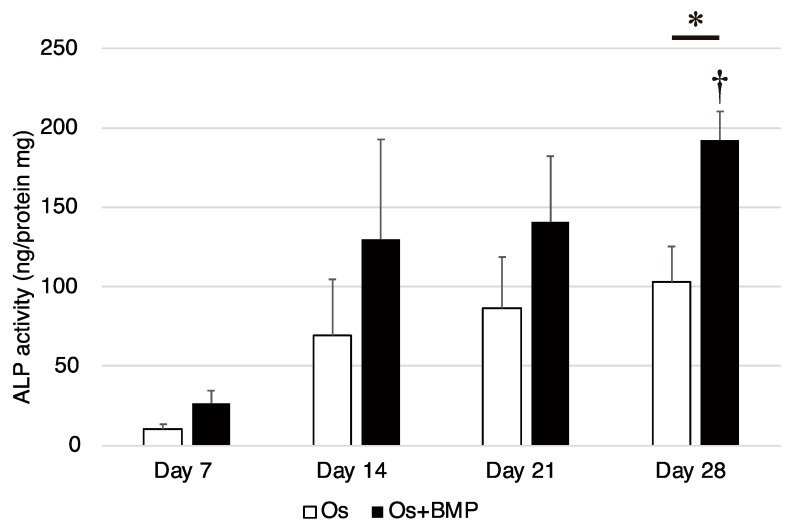
Analysis of the alkaline phosphatase (ALP) activity of non-hypertrophic nonunion cells (NHNCs). ALP activity of NHNCs in osteogenic medium (Os) and Os with E-BMP-2 (Os + BMP) on days 7, 14, 21, and 28. * *p* < 0.05 in the indicated group and ^†^ *p* < 0.05 compared with day 7 results in Os + BMP group.

The ALP activity increased with time in both groups, although there were no significant weekly differences in the Os group (*p* = 0.070). In contrast, the ALP activity was significantly higher on day 28 than on day 7 in the Os + BMP group (*p* = 0.017). In addition, on day 28, the ALP activity in the Os + BMP group was significantly higher than that in the Os group (*p* = 0.016, Figure 4).

**Figure 5:**
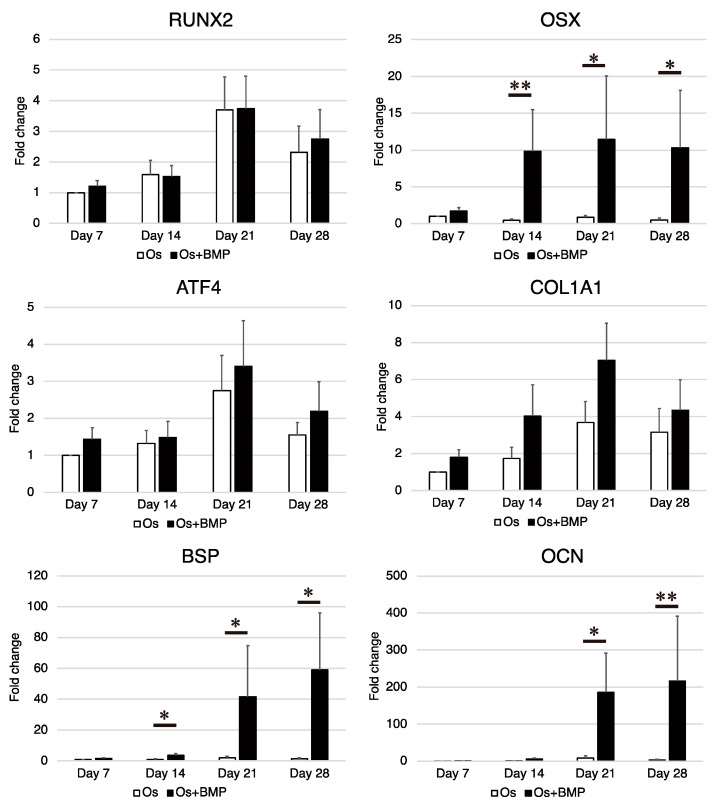
Analysis of the expression of osteoblast-related genes in non-hypertrophic nonunion cells (NHNCs). Real-time reverse transcription-polymerase chain reaction (RT-PCR) for runt-related transcription factor 2 (*RUNX2*), osterix (*OSX*), activating transcription factor 4 (*ATF4*), collagen type I (*COL1A1*), bone sialoprotein (*BSP*), and osteocalcin (*OCN*) in the total RNA extracted from NHNCs in osteogenic medium (Os) and Os with E-BMP-2 (Os + BMP) on days 7, 14, 21, and 28. NHNCs incubated in Os for 7 days were used as the control. * *p* < 0.05 and ** *p* < 0.01 in the indicated groups.

The expression levels of *RUNX2*, *OSX*, *ATF4*, *COL1A1*, *BSP*, and *OCN* were measured using real-time RT-PCR on days 7, 14, 21, and 28 (Figure 5). In the Os group, the expression of *RUNX2*, *ATF4*, and *COL1A1* increased with time and decreased on day 28; however, the levels of *OSX*, *BSP*, and *OCN* were not remarkably upregulated with time. In the Os + BMP group, the expression of *OSX*, *BSP*, and *OCN* was higher than that in the Os group. The difference was significant for *OSX* expression on days 14, 21, and 28 (*p* = 0.009, *p* = 0.047, and *p* = 0.016, respectively), *BSP* expression on days 14, 21, and 28 (*p* = 0.028, *p* = 0.047, and *p* = 0.028, respectively), and *OCN* expression on days 21 and 28 (*p* = 0.028 and *p* = 0.009, respectively). Although the expression of *RUNX2*, *ATF4*, and *COL1A1* in the Os + BMP group was higher than that in the Os group, the differences were not significant.

### 3.4. Chondrogenic Differentiation Potential

**Figure 6:**
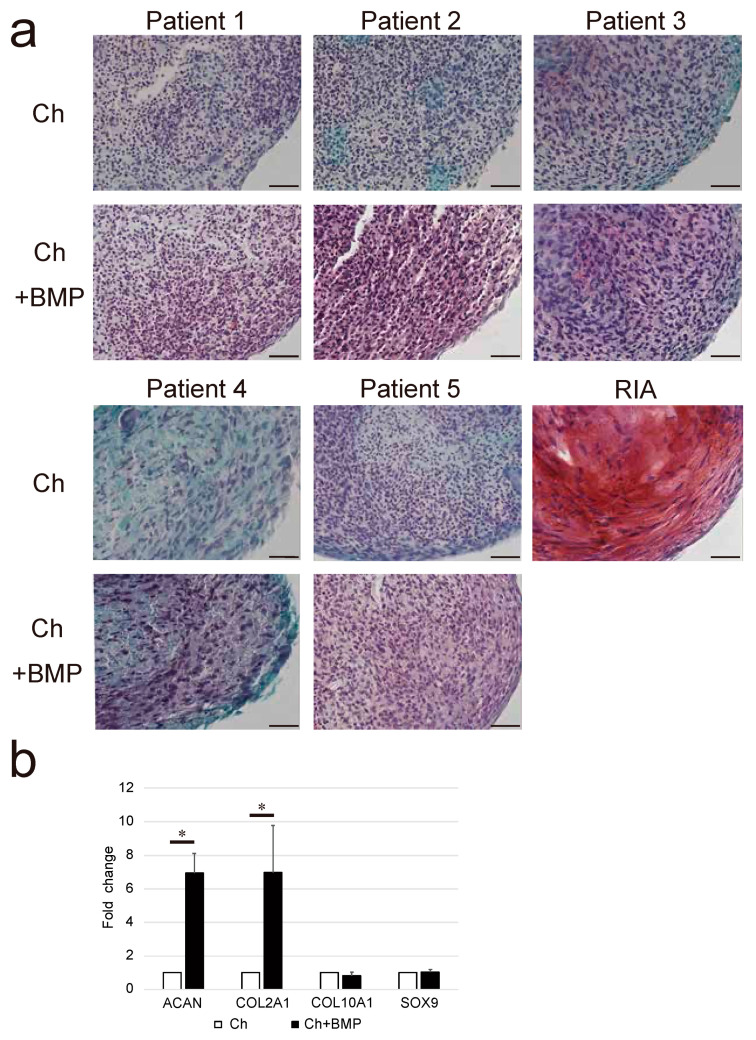
Analysis of the chondrogenic differentiation capability of non-hypertrophic nonunion cells (NHNCs). (**a**) Histological analysis of the chondrogenic capability of NHNCs and cells derived from a reamer-irrigator-aspirator (RIA, positive control) stained using Safranin-O after 21 days of incubation in chondrogenic medium (Ch) and Ch with E-BMP-2 (Ch + BMP). In Ch + BMP, high levels of glycosaminoglycan were deposited and observed as red staining in the extracellular matrix. Scale bars = 50 μm. (**b**) Real-time reverse transcription-polymerase chain reaction (RT-PCR) for aggrecan (*ACAN*), collagen type II (*COL2A1*), collagen type X (*COL10A1*), and Sry-type high-mobility group box 9 (*SOX9*) in the total RNA extracted from NHNC pellets in Ch and Ch + BMP on day 21. NHNCs in Ch were used as control. * *p* < 0.01 in the indicated groups.

Glycosaminoglycan deposition in the Ch + BMP group was observed as red staining of the extracellular matrix by Safranin-O, although this was negligible in the Ch group (Figure 6a). The expression of *ACAN* and *COL2A1* in the Ch + BMP group was significantly higher than that in the Ch group (both *p* = 0.005, Figure 6b). However, the expression of *COL10A1* and *SOX9* did not differ between the groups.

## 4. Discussion

This study demonstrated the ability of NHNCs to proliferate and differentiate into osteoblast-lineage cells, but the cells did not exhibit sufficient chondrogenic differentiation potential. In addition, E-BMP-2 enhanced both osteogenic and chondrogenic differentiation of the cells. In this study, the NHNCs appeared as fibroblast-like spindle-shaped cells resembling bone marrow MSC cells (BMSCs). The proliferation capability of NHNCs was maintained even at passage 10, similar to that of hypertrophic nonunion and pseudoarthrosis tissue-derived cells as previously described [6,7].

Furthermore, the viability of cells in the nonunion fracture site is similar to that of normal BMSCs derived from the human iliac crest [28]. Cells isolated from atrophic fracture nonunion have been reported to exhibit a lower proliferation capability than BMSCs because of increased cell senescence [24]. The NHNCs were actively proliferative at early passages, but this ability decreased slightly during the late passages. The proliferation capability of the NHNCs might have been reduced by an increased level of cell senescence as they were atrophic nonunion-derived cells.

The flow cytometric analysis revealed that the NHNCs exhibited a phenotype similar to that of BMSCs. The capability of NHNCs to differentiate into osteoblast-lineage cells was determined using Alizarin Red S staining, ALP activity analysis, and real-time RT-PCR. The results suggested that the NHNCs have characteristics similar to those of cells from both hypertrophic nonunion and pseudoarthrosis tissues, which have been shown to resemble BMSCs [6,7]. However, the results of Alizarin Red S staining compared with the RIA-derived cells revealed that the NHNCs were less calcified than the BMSCs, which might explain why NHNCs do not show callus formation on X-ray findings [5]. Based on the results of the real-time RT-PCR, we consider that this might also occur because the expression of *OSX*, *BSP*, and *OCN* is not upregulated.

BMP-2 has been widely studied as an osteogenic growth factor and demonstrated to induce bone formation [29,30,31]. However, the effect of BMP-2 treatment on nonunion remains unclear [32]. Zhang et al. [30] reported that BMP-2 causes a dose-dependent decrease in the proliferation of human tendon stem cells derived from the patellar tendons. Our results suggest that the concentration of E-BMP-2 used to treat NHNCs was appropriate as it showed no negative effect on the proliferative capability at each passage.

The differentiation of BMSCs into osteoblasts is mainly mediated by master transcription factors such as RUNX2 and OSX, and BMP-2 stimulates their expression. In our study, E-BMP-2 promoted the osteogenesis of NHNCs by upregulating *OSX* expression; however, it did not upregulate *RUNX2* expression in the NHNCs. We propose that *RUNX2* upregulation in NHNCs by E-BMP-2 might occur before day 7, as BMP-2 upregulates *RUNX2* expression to increase the number of osteoblasts during the early stages of osteoblastic differentiation [33,34].

The chondrogenic differentiation potential of NHNCs was poor, which could be one of the factors involved in the development of non-hypertrophic nonunion. BMP-2 induces chondrogenic differentiation in various types of stem cells in vitro [35]. The results of this study suggest that treating NHNCs with E-BMP-2 induces their differentiation into chondrogenic cells by upregulating ACAN and collagen type II secretion. In contrast, E-BMP-2 treatment did not upregulate the expression of COL10A1 and SOX9, which are transcription factors known to be master regulators of chondrogenesis [36]. We propose that *SOX9* upregulation in NHNCs by E-BMP-2 might occur before day 21. Furthermore, the change in *COL10A1* expression by E-BMP-2 was not detected likely because, as a specific marker for hypertrophic chondrocytes, *COL10A1* is upregulated in the late stages of chondrocyte differentiation. In addition, this observation could further explain why a concentration of 100 ng/mL E-BMP-2 might be insufficient for inducing hypertrophic chondrocytes from NHNCs.

The treatment of hypertrophic nonunion, which is different from that of nonunion, usually requires stabilization of the nonunion site without local treatment such as curettage or bone grafting. This is because the tissue could maintain a reservoir of multilineage mesenchymal progenitor cells, which can transform into cartilage and bone-forming cells [6]. In contrast, the general approach for dealing with most non-hypertrophic nonunion involves improving biological activity by decortication or bone grafting and increasing nonunion site stability [37,38]. Bone autograft is the safest and most effective grafting procedure for nonunion as it uses MSCs from the patient and growth factors to enhance osteogenesis and osteoinduction, respectively [39]. However, the autografts are frequently obtained from the iliac crest, which sometimes causes serious complications such as pain, fractures, bleeding, infection, and nerve palsy in the donor site [40]. These complications might be avoided by the local application of E-BMP-2 without resection of the nonunion tissue.

This study has some limitations. First, our study included only five patients with different fracture sites and different backgrounds. This may cause differences between samples in the activity of mesenchymal stem cells present in non-hypertrophic nonunion tissue. Therefore, further studies with larger samples are required. Second, we think that the NHNCs are mainly composed of MSCs based on the results of flow cytometric analysis. However, considering the complexity around fracture site, we suppose that they were a heterogeneous population of cells with several cell types such as fibroblasts, macrophages, and endothelial cells. Third, we did not directly compare the differentiation and other capabilities of NHNCs to other cells, such as those derived from hypertrophic nonunions or normal human bone marrow mesenchymal stem cells. To determine the actual decrease in the capability of NHNCs, it would be necessary to culture and compare them simultaneously. Finally, we only investigated E-BMP-2 at a concentration of 100 ng/mL, and the effects of varying concentrations need to be examined to determine the appropriate dose.

## 5. Conclusions

We demonstrated for the first time that NHNCs could differentiate into osteoblast-lineage cells, but they did not have a strong calcification or sufficient chondrogenic differentiation capability. Furthermore, E-BMP-2 ameliorated the lack of osteogenic and chondrogenic differentiation potential of NHNCs without affecting their proliferation capability. Local application of E-BMP-2 with the preservation of nonunion tissue is a potentially effective treatment option for non-hypertrophic nonunion through the promotion of osteogenesis and chondrogenesis.

## Figures and Tables

**Table 1:** Cell sample data of the study patients.

Patient	Sex	Age (Years)	Fracture Site	Initial Treatment	Duration from Fracture (Months)
1	Female	20	Clavicle diaphysis	Conservative	6
2	Male	23	Tibia diaphysis	Intramedullary locking nail	10
3	Female	59	Femoral diaphysis	Intramedullary locking nail	9.5
4	Male	24	Femoral diaphysis	Plate-and-screw fixation	12
5	Male	57	Femoral diaphysis	Intramedullary locking nail	12

**Table 2:** Gene-specific primer sequences for real-time reverse transcription-polymerase chain reaction (RT-PCR).

Gene	Primer Sequences
*GAPDH*	Forward: 5′-CGTCTTCACCACCATGGAGA-3′ Reverse: 5′-CGGCCATCACGCCACAGTTT-3′
*RUNX2*	Forward: 5′-ATGCTTCATTCGCCTCACAAAC-3′ Reverse: 5′-CCAAAAGAAGTTTTGCTGACATGG-3′
*OSX*	Forward: 5′-CGGGACTCAACAACTCT-3′ Reverse: 5′-CCATAGGGGTGTGTCAT-3′
*ATF4*	Forward: 5′-CTGACCACGTTGGATGACAC-3′ Reverse: 5′-GGGCTCATACAGATGCCTCT-3′
*COL1A1*	Forward: 5′-AGGAATTCGGCTTCGACGTT-3′ Reverse: 5′-GGTTCAGTTTGGGTTGCTTG-3′
*BSP*	Forward: 5′-AAACGAAGAAAGCGAAGCAGAA-3′ Reverse: 5′-GCTGCCGTTGCCGTTTT-3′
*OCN*	Forward: 5′-CATGAGAGCCCTCACA-3′ Reverse: 5′-AGAGCGACACCCTAGAC-3′
*ACAN*	Forward: 5′-TGAGGAGGGCTGGAACAAGTACC-3′ Reverse: 5′-GGAGGTGGTAATTGCAGGGAACA-3′
*COL2A1*	Forward: 5′-TTTCCCAGGTCAAGATGGTC-3′ Reverse: 5′-CTTCAGCACCTGTCCACCA-3′
*COL10A1*	Forward: 5′-GCCCAAGAGGTGCCCCTGGAATAC-3′ Reverse: 5′-CCTGAGAAAGAGGAGTGGACATAC-3′
*SOX9*	Forward: 5′-ATCTGAAGAAGGAGAGCGAG-3′ Reverse: 5′-TCAGAAGTCTCCAGAGCTTG-3′

*GAPDH*, glyceraldehyde 3-phosphate dehydrogenase; *RUNX2*, runt-related transcription factor 2; *OSX*, osterix; *ATF4*, activating transcription factor 4; *COL1A1*, collagen type I; *BSP*, bone sialoprotein; *OCN*, osteocalcin; *ACAN*, aggrecan; *COL2A1*, collagen type II; *COL10A1*, collagen type X; *SOX9*, Sry-type high-mobility group box 9.

## Data Availability

The datasets used and/or analyzed during the current study are available from the corresponding author on reasonable request.

## Abbreviations

BMP-2	Bone morphogenetic protein-2
E-BMP-2	Escherichia coli-derived bone morphogenetic protein-2
NHNCs	Non-hypertrophic nonunion cells
RT-PCR	Reverse transcription-polymerase chain reaction
rhBMP-2	Recombinant human bone morphogenetic protein-2
CHO	Chinese hamster ovary
PBS	Phosphate-buffered saline
Om	Original medium
PD	Population doubling
Os	Osteogenic medium
ALP	Alkaline phosphatase
RUNX2	Runt-related transcription factor 2
OSX	Osterix
ATF4	Activating transcription factor 4
COL1A1	Collagen type I
BSP	Bone sialoprotein
OCN	Osteocalcin
Ch	Chondrogenic medium
ACAN	Aggrecan
COL2A1	Collagen type II
COL10A1	Collagen type X
SOX9	Sry-type high-mobility group box 9
RIA	Reamer-irrigator-aspirator
MSC	Mesenchymal stromal cell
GAPDH	Glyceraldehyde 3-phosphate dehydrogenase
BMSC	Bone marrow mesenchymal stromal cell

## References

[1] Bishop G.B., Einhorn T.A. (2007). Current and future clinical applications of bone morphogenetic proteins in orthopaedic trauma surgery. Int. Orthop..

[2] Hak D.J., Fitzpatrick D., Bishop J.A., Marsh J.L., Tilp S., Schnettler R., Simpson H., Bishop J.A. (2014). Delayed union and nonunions: Epidemiology, clinical issues, and financial aspects. Injury.

[3] Weber B.G.C.O. (1976). Pseudarthrosis.

[4] Niikura T., Lee S.Y., Sakai Y., Nishida K., Kuroda R., Kurosaka M. (2014). Comparison of radiographic appearance and bone scintigraphy in fracture nonunions. Orthopedics.

[5] Oe K., Zeng F., Fukui T., Nogami M., Murakami T., Matsumoto T., Kuroda R., Niikura T. (2021). Quantitative bone single-photon emission computed tomography imaging for uninfected nonunion: Comparison of hypertrophic nonunion and non-hypertrophic nonunion. J. Orthop. Surg. Res..

[6] Iwakura T., Miwa M., Sakai Y., Niikura T., Lee S.Y., Oe K., Hasegawa T., Kuroda R., Fujioka H., Doita M. (2009). Human hypertrophic nonunion tissue contains mesenchymal progenitor cells with multilineage capacity in vitro. J. Orthop. Res..

[7] Takahara S., Niikura T., Lee S.Y., Iwakura T., Okumachi E., Kuroda R., Kurosaka M. (2016). Human pseudoarthrosis tissue contains cells with osteogenic potential. Injury.

[8] Keskin D.S., Tezcaner A., Korkusuz P., Korkusuz F., Hasirci V. (2005). Collagen-chondroitin sulfate-based PLLA-SAIB-coated rhBMP-2 delivery system for bone repair. Biomaterials.

[9] Zara J.N., Siu R.K., Zhang X., Shen J., Ngo R., Lee M., Li W., Chiang M., Chung J., Kwak J. (2011). High doses of bone morphogenetic protein 2 induce structurally abnormal bone and inflammation in vivo. Tissue Eng. Part A.

[10] Fung S.L., Wu X., Maceren J.P., Mao Y., Kohn J. (2019). In vitro evaluation of recombinant bone morphogenetic Protein-2 bioactivity for regenerative medicine. Tissue Eng. Part C Methods.

[11] Israel D.I., Nove J., Kerns K.M., Moutsatsos I.K., Kaufman R.J. (1992). Expression and characterization of bone morphogenetic protein-2 in Chinese hamster ovary cells. Growth Factors.

[12] Lee J.H., Jang S.J., Koo T.Y., Suh C.W., Lee E.N., Lee K.M., Lee H.S., Baek H.R. (2011). Expression, purification and osteogenic bioactivity of recombinant human BMP-2 derived by *Escherichia coli*. J. Tissue Eng. Regen. Med..

[13] Long S., Truong L., Bennett K., Phillips A., Wong-Staal F., Ma H. (2006). Expression, purification, and renaturation of bone morphogenetic protein-2 from *Escherichia coli*. Protein Expr. Purif..

[14] Yano K., Hoshino M., Ohta Y., Manaka T., Naka Y., Imai Y., Sebald W., Takaoka K. (2009). Osteoinductive capacity and heat stability of recombinant human bone morphogenetic protein-2 produced by *Escherichia coli* and dimerized by biochemical processing. J. Bone Miner. Metab..

[15] Kuroiwa Y., Niikura T., Lee S.Y., Oe K., Iwakura T., Fukui T., Matsumoto T., Matsushita T., Nishida K., Kuroda R. (2019). Escherichia coli-derived BMP-2-absorbed beta-TCP granules induce bone regeneration in rabbit critical-sized femoral segmental defects. Int. Orthop..

[16] Megas P. (2005). Classification of non-union. Injury.

[17] Hernigou P., Poignard A., Beaujean F., Rouard H. (2005). Percutaneous autologous bone-marrow grafting for nonunions. Influence of the number and concentration of progenitor cells. J. Bone Jt. Surg. Am..

[18] Oe K., Miwa M., Sakai Y., Lee S.Y., Kuroda R., Kurosaka M. (2007). An in vitro study demonstrating that haematomas found at the site of human fractures contain progenitor cells with multilineage capacity. J. Bone Jt. Surg. Br..

[19] Ruppert R., Hoffmann E., Sebald W. (1996). Human bone morphogenetic protein 2 contains a heparin-binding site which modifies its biological activity. Eur. J. Biochem..

[20] Matsumoto T., Toyoda H., Dohzono S., Yasuda H., Wakitani S., Nakamura H., Takaoka K. (2012). Efficacy of interspinous process lumbar fusion with recombinant human bone morphogenetic protein-2 delivered with a synthetic polymer and beta-tricalcium phosphate in a rabbit model. Eur. Spine J..

[21] Cristofalo V.J., Allen R.G., Pignolo R.J., Martin B.G., Beck J.C. (1998). Relationship between donor age and the replicative lifespan of human cells in culture: A reevaluation. Proc. Natl. Acad. Sci. USA.

[22] Maeda T., Matsunuma A., Kurahashi I., Yanagawa T., Yoshida H., Horiuchi N. (2004). Induction of osteoblast differentiation indices by statins in MC3T3-E1 cells. J. Cell Biochem..

[23] Lee S.Y., Miwa M., Sakai Y., Kuroda R., Matsumoto T., Iwakura T., Fujioka H., Doita M., Kurosaka M. (2007). In vitro multipotentiality and characterization of human unfractured traumatic hemarthrosis-derived progenitor cells: A potential cell source for tissue repair. J. Cell Physiol..

[24] Bajada S., Marshall M.J., Wright K.T., Richardson J.B., Johnson W.E. (2009). Decreased osteogenesis, increased cell senescence and elevated Dickkopf-1 secretion in human fracture non union stromal cells. Bone.

[25] Schmidmaier G., Herrmann S., Green J., Weber T., Scharfenberger A., Haas N.P., Wildemann B. (2006). Quantitative assessment of growth factors in reaming aspirate, iliac crest, and platelet preparation. Bone.

[26] Kuehlfluck P., Moghaddam A., Helbig L., Child C., Wildemann B., Schmidmaier G., HTRG-Heidelberg Trauma Research Group (2015). RIA fractions contain mesenchymal stroma cells with high osteogenic potency. Injury.

[27] Toosi S., Naderi-Meshkin H., Kalalinia F., Peivandi M.T., Hossein K.H., Bahrami A.R., Heirani-Tabasi A., Mirahmadi M., Behravan J. (2016). Comparative characteristics of mesenchymal stem cells derived from reamer-irrigator-aspirator, iliac crest bone marrow, and adipose tissue. Cell Mol. Biol..

[28] Ismail H.D., Phedy P., Kholinne E., Kusnadi Y., Sandhow L., Merlina M. (2013). Existence of mesenchymal stem cells in sites of atrophic nonunion. Bone Jt. Res..

[29] Geiger M., Li R.H., Friess W. (2003). Collagen sponges for bone regeneration with rhBMP-2. Adv. Drug Deliv. Rev..

[30] Zhang J., Wang J.H. (2012). BMP-2 mediates PGE_2_-induced reduction of proliferation and osteogenic differentiation of human tendon stem cells. J. Orthop. Res..

[31] Gromolak S., Krawczenko A., Antończyk A., Buczak K., Kiełbowicz Z., Klimczak A. (2020). Biological characteristics and osteogenic differentiation of ovine bone marrow derived mesenchymal stem cells stimulated with FGF-2 and BMP-2. Int. J. Mol. Sci..

[32] Garrison K.R., Shemilt I., Donell S., Ryder J.J., Mugford M., Harvey I., Song F., Alt V. (2010). Bone morphogenetic protein (BMP) for fracture healing in adults. Cochrane Database Syst. Rev..

[33] Kämmerer P.W., Pabst A.M., Dau M., Staedt H., Al-Nawas B., Heller M. (2020). Immobilization of BMP-2, BMP-7 and alendronic acid on titanium surfaces: Adhesion, proliferation and differentiation of bone marrow-derived stem cells. J. Biomed. Mater Res. A.

[34] Komori T. (2010). Regulation of bone development and extracellular matrix protein genes by RUNX2. Cell Tissue Res..

[35] Zhou N., Li Q., Lin X., Hu N., Liao J.Y., Lin L.B., Zhao C., Hu Z.-M., Liang X., Xu W. (2016). BMP2 induces chondrogenic differentiation, osteogenic differentiation and endochondral ossification in stem cells. Cell Tissue Res..

[36] Hino K., Saito A., Kido M., Kanemoto S., Asada R., Takai T., Cui M., Cui X., Imaizumi K. (2014). Master regulator for chondrogenesis, Sox9, regulates transcriptional activation of the endoplasmic reticulum stress transducer BBF2H7/CREB3L2 in chondrocytes. J. Biol. Chem..

[37] Tall M., Bonkoungou D., Sawadogo M., Da S.C., Toe M.F. (2014). Treatment of nonunion in neglected long bone shaft fractures by osteoperiosteal decortication. Orthop. Traumatol. Surg. Res..

[38] Sen M.K., Miclau T. (2007). Autologous iliac crest bone graft: Should it still be the gold standard for treating nonunions?. Injury.

[39] Caplan A.I. (1991). Mesenchymal stem cells. J. Orthop. Res..

[40] Gómez-Barrena E., Padilla-Eguiluz N.G., Rosset P. (2020). Frontiers in non-union research. EFORT Open Rev..

